# Refractory Bradycardia and Unreactive Mydriasis Revealing Severe Intraoperative Neonatal Hypothermia in a Resource-Limited Setting

**DOI:** 10.7759/cureus.87931

**Published:** 2025-07-14

**Authors:** Sai Servais Sontia, Koffi Koffi Maxime, Coulibaly Klinna Theodore, Thomas Helen Audrey, Moulot Martial Olivier

**Affiliations:** 1 Intensive Care Unit, Treichville University Hospital, Abidjan, CIV; 2 Pediatric Surgery, Felix Houphouët Boigny University, Abidjan, CIV; 3 Anesthesia, Intensive Care, and Emergency Medicine, Felix Houphouët Boigny University, Abidjan, CIV; 4 Pediatric Surgery, Treichville University Hospital, Abidjan, CIV

**Keywords:** intraoperative neonate hypothermia, paediatric anaesthesia, refractory bradycardia, resource-limited settings, unreactive mydriasis

## Abstract

Perioperative hypothermia represents a major complication in pediatric anesthesia, particularly in neonates. We report the case of a newborn who underwent general anesthesia for esophageal fistula repair (following primary repair of esophageal atresia) and developed severe hypothermia manifested by the triad of refractory severe bradycardia, bilateral areflexic mydriasis, and chest wall rigidity. Management combining active rewarming, alkalinization, and cardiovascular support resulted in a complete recovery with a progressive normalization of clinical parameters. We discuss the clinical signs observed during this complication and draw the attention of anesthesiologists practicing in resource-limited settings, for whom these signs may enable early diagnosis of this complication.

## Introduction

Perioperative hypothermia is a historically recognized complication of anesthesia [1]. It is defined as a reduction in core body temperature below 36.5°C [2] and can be classified into three stages according to severity: mild (32-35°C), moderate (28-32°C), and severe (<28°C) [3]. Clinical manifestations vary according to the degree of severity and are associated with multiple complications. Most commonly in the initial stage, shivering, tachycardia, and even altered consciousness are observed. During the perioperative period, bradycardia and ECG changes (J waves) have been described [3].

In low-income countries, perioperative hypothermia poses a significant public health challenge [4]. The contributing factors include ineffective thermal control in operating theaters, restricted access to warming devices or their misuse, prolonged surgical durations often due to equipment shortages, and prevalent patient malnutrition [2]. In this context, intraoperative hypothermia detection is complicated by the absence of continuous temperature monitoring. During general anesthesia, impaired higher functions may delay the diagnosis of this complication, which is often identified at an advanced stage. Knowledge of clinical signs suggestive of intraoperative hypothermia is therefore fundamental for anesthetists practicing in resource-limited settings. Neonates, who represent the most vulnerable population to this complication (high mortality risk), warrant particular attention given the mortality implications [2]. Few reports describe the neurological or cardiovascular manifestations of intraoperative hypothermia in neonates.

In this article, we present a case of severe intraoperative hypothermia occurring in a neonate without continuous temperature monitoring. The objective is to share the severity of clinical signs observed during this severe accidental hypothermia and to explain their underlying mechanisms. This clinical case has not been previously presented at any scientific meeting.

## Case presentation

A female neonate, aged 17 days (weight: 3.8 kg), had undergone surgery for esophageal atresia on day 8 of life. The initial surgery proceeded without incident under general anesthesia with insertion of a right-sided chest drain. The postoperative course was complicated by a postoperative fistula. A second surgical procedure was therefore indicated to repair the fistula, perform an esophagostomy, and create a feeding gastrostomy.

Preoperative anesthetic assessment noted a satisfactory general condition in the 17-day-old infant, who was afebrile with stable cardiorespiratory parameters. Respiratory examination revealed a normal respiratory rate (42 breaths per minute) without signs of respiratory distress, oxygen saturation between 89% and 95% (on 3L via nasal cannula), and right-sided bronchial rales, with a right chest drain under suction. Laboratory investigations showed mild hypernatremia, anemia, and elevated creatinine (Table 1). The American Society of Anesthesiologists (ASA) score was assessed as 3.

**Table 1:** Summary of preoperative laboratory workup

Parameter	Values	Normal Range
White blood cells (cells/mm³)	12,040	8.000–21,000
Hemoglobin (g/dl)	13.1	15.5–20.5
Hematocrit (%)	40.2	47.0–65.0
Platelets (cells/mm³)	394,000	150,000–400,000
Serum creatinine (mg/l)	18.7	2.4-5.2
Blood urea nitrogen (g/l)	1.12	0.15−0.45
Serum sodium (mmol/l)	150.8	137–145
Serum potassium (mmol/l)	5.48	3.50–5.50
Serum chloride (mmol/L)	119.2	95.0–110.0
Serum calcium (mg/l)	91.80	80–112
C-reactive protein (mg/l)	32	<6
Blood group	B positive	-

The patient was admitted to the operating theater with an ambient temperature of 20°C and positioned on an operating table equipped with a heating mattress. Multiparametric monitoring included non-invasive blood pressure measurement, electrocardiography, and pulse oximetry. Anesthetic induction with propofol (20 mg) and fentanyl (4 μg) facilitated easy intubation (2.5 mm tracheal tube). The patient was ventilated using volume-controlled mechanical ventilation. Anesthetic maintenance was achieved with sevoflurane adjusted according to minimum alveolar concentration (MAC, 1-1.5%). Fluid management included 10% dextrose enriched with calcium and potassium.

After approximately 190 minutes of surgery, progressive bradycardia developed. Given normal respiratory parameters and absence of hypoxia, anesthetic overdose was suspected. Sevoflurane administration was therefore discontinued. Transfusion of 50 ml ABO and Rh-compatible packed red blood cells was initiated. More than 45 minutes after discontinuing the volatile agent, bradycardia worsened (approximately 80 bpm) and persisted despite atropine administration. The patient exhibited bilateral unreactive mydriasis. Capillary blood glucose was 1.41 g/L. Subsequently, thoracic rigidity developed, associated with gasping respirations. No cyanosis or signs of peripheral hypoperfusion were noted. Manual ventilation was continued.

In the absence of a temperature probe on the monitor, core body temperature measurement with an infrared thermometer revealed severe hypothermia at 27.6°C (despite the presence of the heating mattress). Laboratory investigations revealed severe acidosis (Table 2). Values that could not be calculated by blood gas analysis were due to the fact that the parameters analyzed were too low.

**Table 2:** Arterial blood gas analysis obtained during surgery pH=potential of hydrogen; pCO₂=partial pressure of carbon dioxide; pO₂=partial pressure of oxygen; Total CO₂=temperature-corrected CO₂; Base excess=metabolic acid-base component; Hematocrit=packed cell volume. *NC = not calculable

Parameter	Values	Normal Range
pH	<6.8	7.35–7.45
pCO₂ (mmHg)	32	35–45
pO₂ (mmHg)	209	75–100
HCO₃⁻ (mmol/l)	*NC	22–26
Total CO₂ (mmol/l)	*NC	23–27
Base excess	*NC	-2 to +2
Lactate (mmol/l)	0.92	0.90–1.70
Na⁺ (mmol/l)	146	137–145
K⁺ (mmol/l)	4.7	3.50–5.50
Glucose (mmol/l)	8.4	3.9–6.1
Hematocrit (%)	42	47.0–65.0
Ca²⁺ (mmol/l)	1.25	1.15–1.35

Given normal respiratory parameters (EtCO₂, SpO₂), the acidosis was presumed to be metabolic in origin, and urgent alkalinization was undertaken (20 ml bicarbonate bolus followed by 20 ml continuous infusion over three hours). The patient also received titrated adrenaline (three doses of 0.1 ml at a dose of 0.1 ml/kg of 1:10,000 solution), which progressively achieved a heart rate >113 bpm. We simultaneously enhanced physical warming measures (using external warming devices and warm bottles applied to axillary and inguinal regions).

The clinical course was marked by progressive recovery of effective spontaneous ventilation and gradual regression of bilateral mydriasis. Approximately 1 hour post-surgery, voluntary movements returned with spontaneous eye opening and yawning around the endotracheal tube. The child was transferred to the neonatal intensive care unit postoperatively, exhibiting a Blantyre coma score of 5 upon admission, an admission temperature of 36.8°C and capillary blood glucose of 1.35 g/l. Mechanical ventilation was discontinued at 18 hours post-surgery following correction of acidosis. The postoperative course was satisfactory and the patient was discharged after a total length of stay of 20 days.

## Discussion

This article presents a case of severe hypothermia occurring during thoraco-abdominal surgery in a female neonate patient under general anesthesia. The delayed diagnosis revealed uncommon clinical signs typically observed only in severe forms of hypothermia. This observation is valuable for anesthetists practicing in resource-limited areas (absence of intraoperative temperature monitoring) for early diagnosis of this complication. In our case, refractory bradycardia (without arterial hypotension), unreactive mydriasis, thoracic rigidity, and metabolic acidosis particularly attracted our attention. We describe the pathophysiological mechanisms underlying these signs.

In pediatric anesthesia, bradycardia in children most commonly results from hypoxemia or volatile agent overdose [5]. In our case, it occurred despite adequate oxygenation and worsened despite sevoflurane discontinuation. It results from the deleterious effects of hypothermia on cardiac automaticity and conduction (slowing of sinoatrial node depolarization, reduced kinetics of Na⁺/K⁺ ion channels, and decreased cardiac responsiveness to catecholamines) [6-9]. As observed in our patient, this bradycardia is characterized by its refractory nature to atropine, as it is not vagally mediated but related to metabolic depression [6-9]. Blood pressure remains preserved for extended periods due to a compensatory increase in stroke volume. Other cardiac manifestations such as J waves or arrhythmias may be observed [8].

Severe hypothermia causes metabolic acidosis (through acid overproduction) due to concurrent mitochondrial dysfunction and increased anaerobic metabolism [8-10]. Beyond acid production, cold inhibits both respiratory and renal compensatory mechanisms for acidosis. Respiratory compensation is impaired as hypothermia alters ventilatory response to acidosis, promotes thoracic rigidity, and shifts the hemoglobin dissociation curve leftward, preventing respiratory compensatory mechanisms. Regarding renal compensation, there is decreased tubular bicarbonate reabsorption and reduced H⁺ ion excretion [10]. Excluding acidosis, the remaining laboratory abnormalities (hypernatremia, renal dysfunction, and anemia) were unrelated to the hypothermia. Both hypernatraemia and acute renal injury resulted from pathology-induced dehydration, while the anemia was likely attributable to the patient's nutritional status.

These two phenomena (acidosis and bradycardia) perpetuate each other in a pathological vicious cycle. Bradycardia may cause low cardiac output with organ hypoperfusion, leading to anaerobic metabolism and acidosis. Conversely, acidosis causes myocardial depression, promoting bradycardia and organ hypoperfusion.

Opioids are the most implicated agents in anesthesia-related thoracic rigidity. In our case, fentanyl was administered only during anesthetic induction, and thoracic rigidity was observed more than four hours after surgery commencement (well after fentanyl injection). Severe hypothermia causes skeletal muscle rigidity. At the thoracic level, cold reduces thoracic compliance through various mechanisms: altered elasticity of cartilaginous and ligamentous structures, permanent tonic contracture of intercostal muscles, and increased viscosity of thoracic connective tissue [8,9]. The consequence is increased work of breathing (causing gasping in our patient) and hypoventilation.

Bilateral unreactive mydriasis may be observed in severe hypothermia (<28°C) [9,11,12]. This phenomenon is primarily neurological, related to sympatho-parasympathetic imbalance. During hypothermia, progressive inhibition of mesencephalic centers impairs parasympathetic response (pupillary vasoconstriction). Thus, sympathetic dilator action becomes predominant through α1 adrenergic receptor stimulation during the initial stress phase of hypothermia.

In sub-Saharan Africa, and specifically in our country, data on perioperative hypothermia are scarce. In some low-income countries, prevalence has been estimated at 75% [13]. The risk factors for intraoperative hypothermia are well established [13]. In neonates, these include immature thermoregulatory mechanisms, reduced weight-to-body surface area ratio, increased heat loss from the head, and limited subcutaneous fat reserves [2]. Beyond our patient's young age, procedural duration (approximately five hours) and intervention type (thoraco-abdominal) were additional surgery-related risk factors. Regarding environmental factors, this experience demonstrates that heating mattresses cannot serve as the sole means of preventing intraoperative hypothermia in infants. Other measures such as warmed solutions, appropriate ambient temperature regulation, and external warming systems (in addition to heating mattresses) during surgery must be employed to prevent this complication [2,13].

## Conclusions

The clinical significance of perioperative hypothermia is frequently underappreciated in low- and middle-income countries, where it represents a preventable cause of increased pediatric morbidity and mortality. The clinical importance of perioperative hypothermia is often underestimated in low- and middle-income countries, where it represents a preventable cause of increased paediatric morbidity and mortality. Its prevention requires continuous temperature monitoring and the use of external warming devices. In the absence of these tools, the unexpected occurrence in a neonate of refractory bradycardia, bilateral mydriasis, J waves on the ECG, or chest wall rigidity represents warning signs for the anesthetist.
